# Comparing the hemodynamic effect of a large arteriovenous fistula during high and low cardiac output states

**DOI:** 10.3389/fphys.2023.1180224

**Published:** 2023-07-03

**Authors:** A. Valerianova, M. Mlcek, J. Malik, T. Grus, L. Tejkl, B. Kolosova, V. Lejsek, O. Kittnar

**Affiliations:** ^1^ 3rd Department of Internal Medicine, General University Hospital in Prague and 1st Faculty of Medicine, Charles University, Prague, Czechia; ^2^ Institute of Physiology, 1st Faculty of Medicine, Charles University, Prague, Czechia; ^3^ 2nd Surgical Clinic—Cardiovascular Surgery, General University Hospital in Prague and 1st Faculty of Medicine, Charles University, Prague, Czechia

**Keywords:** arteriovenous fistula, hyperkinetic circulation, dobutamine, norepinephrine, low cardiac output

## Abstract

**Background:** A large arteriovenous fistula (AVF) is a low-resistant circuit that affects organ perfusion and systemic hemodynamics even in standard conditions. The extent of its’ effect in critical states has not been elucidated yet. We used norepinephrine to create systemic vasoconstriction, dobutamine to create high cardiac output, and rapid right ventricle pacing as a model of acute heart failure in a porcine model of high-flow AVF circulation.

**Methods:** The protocol was performed on nine domestic female pigs under general anesthesia. AVF was created by connecting two high-diameter ECMO cannulas inserted in the femoral artery and vein. Continuous hemodynamic monitoring was performed throughout the protocol. Three interventions were performed–moderate dose of norepinephrine (0.25 ug/kg/min), moderate dose of dobutamine (10 ug/kg/min) and rapid right ventricle pacing to simulate low cardiac output state with mean arterial pressure under 60 mmHg. Measurements were taken with opened and closed arteriovenous fistula.

**Results:** Continuous infusion of norepinephrine with opened AVF significantly increased mean arterial pressure (+20%) and total cardiac output (CO) (+36%), but vascular resistance remained virtually unchanged. AVF flow (Qa) rise correlated with mean arterial pressure increase (+20%; R = 0.97, *p* = 0.0001). Effective cardiac output increased, leading to insignificant improvement in organ perfusion. Dobutamine substantially increased cardiac output with insignificant effect on AVF flow and mean arterial pressure. Carotid artery blood flow increased significantly after dobutamine infusion by approximately 30%, coronary flow velocity increased significantly only in closed AVF state. The effective cardiac output using the heart failure model leading to decrease of carotid artery flow and worsening of brain and peripheral tissue oximetry. AVF blood flow also dropped significantly and proportionally to pressure, but Qa/CO ratio did not change. Therefore, the effective cardiac output decreased.

**Conclusion:** In abovementioned extreme hemodynamic conditions the AVF flow was always directly proportional to systemic perfusion pressure. The ratio of shunt flow to cardiac output depended on systemic vascular resistance. These experiments highlight the detrimental role of a large AVF in these critical conditions’ models.

## Introduction

A large arteriovenous fistula (AVF) is a low-resistant circuit that can substantially affect the hemodynamics and organ perfusion since a significant part of the cardiac output (10%–40% in humans) is shunted from systemic arteries to veins. The term effective cardiac output represents the difference of total cardiac output and AVF flow (Basile et al., 2007). The detrimental effect of the AVF on brain perfusion and tissue oxygenation, or on transplanted kidney perfusion has been described previously in humans (Laranjinha et al., 2019; Kovarova et al., 2021). Our working group has already created an animal model of hyperkinetic circulation with the use of a high flow AVF. However, patients with AVFs of any origin can be exposed to different critical conditions requiring inotropic or vasopressor support, or states with sudden increase or decrease in cardiac output (e.g., sepsis, acute heart failure, etc.). The extent of the steal of systemic circulation by AVF is not known under such conditions.

Dobutamine is a positive inotropic agent directly stimulating the β_1_ adrenergic receptors. It increases cardiac output (CO) by increased cardiac contractility, leading to increased coronary artery blood flow. Reflexively, systemic vascular resistance is decreased. Therefore, dobutamine has no or small effect on systemic arterial pressure in humans (Kern, 2003; Dubin et al., 2017). It is one of the most frequently used medicaments for increasing CO in heart failure including cardiogenic shock.

Norepinephrine is a potent vasopressor indicated to increase arterial blood pressure in acute hypotension during shock states of different etiology. It is a sympathomimetic drug stimulating the α adrenergic receptors (responsible for vasoconstrictive effect) and β_1_ receptors (responsible for positive inotropic effect) (Hollenberg, 2011).

Rapid right ventricular (RV) pacing is a frequently used animal model of heart failure (Ogilvie and Zborowska-Sluis, 1992; Li et al., 2006). The acute effects of cardiac output decrease manifest shortly after the start of pacing. Other effects including neurohumoral activation and typical development of tachycardia-induced cardiomyopathy need more time (approximately weeks) to develop. Besides that, a log-term large AVF can induce development of heart failure–a mechanism known from both small biomodels (Petrak et al., 2011; Malik et al., 2021) and human medicine.

The aim of this study was to quantify the effect of a large AVF during norepinephrine or dobutamine therapy in a large animal model. In this series of experiments, we aimed to describe the effect of AVF during the following states: a) high cardiac output state caused by continuous dobutamine infusion, b) systemic vasoconstriction induced by continuous norepinephrine infusion, c) acute heart failure with low cardiac output induced by fast RV stimulation. Our hypotheses were: 1. norepinephrine will increase AVF flow with higher systemic arterial pressure at the cost of tissue perfusion worsening; 2. Dobutamine-mediated increase in cardiac output will be substantially stolen by the AVF; 3. In the model of heart failure, AVF will contribute to worsening of organ perfusion.

## Materials and methods

The study was performed in The Common Experimental Laboratory of the Department of Physiology, First Faculty of Medicine, Charles University, Prague, on female domestic pigs. The animals were handled in accordance with guidelines and legal requirements for animal use in research. The study was approved by Institutional care and use committee and was performed in accordance with European Guidelines on Laboratory Animal Care.

### The protocol

The experiments were performed on female domestic swine (*Sus scrofa domestica*). All the experiments were held under general anaesthesia. After premedication with intramuscular (i. m.) injection of midazolam (0.3 mg/kg) and ketamine (20 mg/kg), the marginal ear vein was cannulated. After preoxygenation with 100% oxygen via a facial mask, the general anaesthesia was induced with intravenous bolus (i.v.) of propofol (1–2 mg/kg) followed by a continuous infusion. The orotracheal intubation was performed and mechanical ventilation was started. During the procedure, the ventilation was adjusted to maintain normoxia (peripheral oxygen saturation ≥ 97%, pO_2_ 100 mmHg) and normocapnia (EtCO_2_ 38–40 mmHg, pCO_2_ 35–45 mmHg). During the whole experiment total intravenous anaesthesia was maintained by continuous intravenous infusion of propofol, morphine and midazolam with regular controls of pupillary and corneal reflexes to adjust the depth of anaesthesia. Doses of anaesthetics remained unchanged during the protocol to avoid the effect of dose variation on brain perfusion and cardiovascular system. Anticoagulation was maintained by unfractionated heparin, starting with i.v. bolus 100 units per kilogram followed by continuous i.v. infusion to reach the target activated clotting time (200–250 s). Initial fluid bolus of 1,000 mL was followed by continuous i.v. infusion to maintain central venous pressure between 6–8 mmHg.

Sheaths and catheters were inserted into the jugular vein and brachial arteries and measurement probes and catheters were inserted as described in section *Measured parameters*. Pacing catheter was inserted into the apex of the right ventricle for rapid stimulation to create the low cardiac output state.

The AVF was created by a connection of femoral artery and femoral vein using percutaneously inserted high diameter ECMO cannulas (18 F for the arterial cannula and 22 F for venous cannula) as described previously (Valerianova et al., 2022). AVF blood flow was continuously measured by transient-time ultrasound probe fixed to the ECMO set (Transonic, United States). A special clamp fixed around the arterial cannula was used to regulate the AVF blood flow and to close the fistula when required. Initial AVF flow measurement was performed to verify a sufficient AVF functioning and then continued during the whole experiment.

**FIGURE 1:**
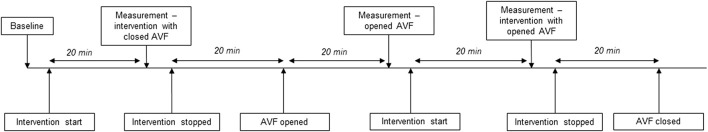
Scheme of study protocol.The protocol for the interventions was performed as depicted in the scheme. New baseline was set before each intervention. For heart failure, the times of “intervention” were shortened to 10 min to prevent further hemodynamic deterioration.

The protocol is summarized in Figure 1. New baseline measurement was performed before start of each intervention. Norepinephrine was administered as a continuous intravenous infusion, dose 0.25 μg/kg/min. Dobutamine was administered as continuous intravenous infusion, dose 10 μg/kg/min. The protocol was identical for both interventions. After baseline data collection, continuous infusion of dobutamine or norepinephrine was started. After 20 min of stabilization, hemodynamic measurements were collected. Then the infusion was paused for approximately 20 min until the vital parameter values returned to baseline. Afterwards the AVF was opened and AVF data was collected. Infusion with medication was then resumed with opened AVF and data was collected approximately after 20 min of stabilization. For low CO protocol, rapid right ventricular pacing was induced via pacing electrode inserted into the right ventricle under skiascopic control. Stimulation rate was set to achieve stable mean arterial pressure below 60 mmHg. Parameters with closed and opened AVF were recorded after 10 min of continuous rapid pacing.

### Measured parameters

Continuous surface ECG, peripheral oxygen saturation and, EtCO_2_ monitoring were performed. Continuous invasive arterial blood pressure was measured using a monitoring catheter inserted in the left radial artery. Central venous pressure was measured using the central venous catheter inserted via the left jugular artery. Pulmonary arterial pressure, continuous measurement of mixed venous oximetry (SvO_2_) and cardiac output measured by thermodilution method were recorded using the Swan-Ganz catheter (CCOmbo, Edwards Lifesciences) inserted deep into the pulmonary artery under X-ray control via the right jugular vein. Carotid artery flow was continuously measured by transient-time perivascular ultrasound probe (Transonic, United States). The intracoronary artery flow velocity was measured by a Doppler flow wire (FloWire, Volcano, United States) inserted into the left anterior descending or circumflex artery approximately 8 cm distal to the left coronary artery ostium. Measurement of regional tissue oxygen saturation was performed using the INVOS 5100C oximetry system (Medtronic), working on the near-infrared spectroscopy principle. The sensors were placed on the top of the head and on the front leg.

### Calculated parameters

Mean arterial pressure (MAP) was calculated as MAP = 2/3*ART(d) + 1/3*ART(s) (ART–systemic arterial pressure, (d)—diastolic, (s)—systolic). Stroke volume (SV) was calculated as SV = CO/HR (CO–cardiac output, HR–heart rate). Effective cardiac output (COeff) vas calculated as CO minus AVF flow. Left ventricular stroke work was approximated as *SVx(MAP-PCW)x0.0136* (SV–stroke volume, MAP–mean arterial pressure, PCW–pulmonary capillary wedge pressure). In analogy, right ventricular stroke work was calculated as *SVx(PAMP-CVP)x0.0136* (PAM–pulmonary artery mean pressure, CVP–central venous pressure). Total vascular resistance (TVR), systemic vascular resistance (SVR) and pulmonary vascular resistance (PVR) were calculated: TVR = (MAP-CVP)/CO (MAP–mean arterial pressure, CVP–central venous pressure, CO–cardiac output), SVR = (MAP-CVP)/COeff (MAP–mean arterial pressure, CVP–central venous pressure, COeff–effective cardiac output), PVR = (PAMP-PCWP)/CO (PAMP–pulmonary artery mean pressure, PCWP–pulmonary capillary wedge pressure, CO–cardiac output).

### Data collection and statistics

All the data was real-time recorded and stored on a PC using ADI PowerLab ADC and LabChart Pro software (ADInstruments, New Zealand).

The data was analyzed using the Statistica software (StatSoft Inc., United States). Paired *t*-test or ANOVA were used as appropriate. Continuous variables are presented as mean ± SD. The *p*-value < 0.05 was considered statistically significant.

## Results

Nine healthy swine were included into the study, weighing 62 ± 5 kg. The predefined interventions were performed in different order in each animal. One animal died of hemorrhagic shock after first two procedures (norepinephrine and dobutamine administration). Therefore, the heart failure protocol was performed only in 8 animals.

### Norepinephrine

Initiation of norepinephrine infusion led to the increase of mean arterial pressure both with and without patent AVF; the increase was more pronounced with closed AVF. Detailed results are presented in Table 1. The response of cardiac output was not consistent. In closed AVF, cardiac output increased in 5 animals and slightly decreased or remained unchanged in 4 animals. With AVF opened, cardiac output increased in all animals except one. AVF flow increased by 20% with norepinephrine. The increase in AVF flow positively correlated with increase in MAP (R = 0.97; *p* = 0.0001). Although Qa/CO ratio did not change significantly with norepinephrine, the effective cardiac output increased. COeff did not correlate with MAP during norepinephrine administration (R = 0.227). LV work increased after norepinephrine in both closed and opened AVF states.

**TABLE 1:** Effect of continuous norepinephrine infusion.

Parameter	Closed AVF		Opened AVF	
	Without norepinephrine	With norepinephrine	Without norepinephrine	With norepinephrine
CO (L/min)	8.0 ± 1.5	8.7 ± 2.7	8.2 ± 3.2	10.7 ± 2.5**
Qa (L/min)			2.0 ± 0.4	2.4 ± 0.6
COeff (L/min)			6.2 ± 2.8	8.3 ± 2.3**
Qa/CO (%)			26.3 ± 5.9	23.1 ± 6.0
HR (/min)	111 ± 33	119 ± 36	129 ± 49	143 ± 46
SV (mL)	77 ± 21	79 ± 31	69 ± 21	82 ± 26
MAP (mmHg)	84 ± 14	116 ± 23**	79 ± 21	105 ± 22*
PAMP (mmHg)	26 ± 8	37 ± 14**	28 ± 12	34 ± 16
SvO_2_ (%)	60 ± 9	65 ± 15	70 ± 12	67 ± 14
TVR (WU)	10.3 ± 2.7	13.6 ± 4.5*	9.7 ± 4.0	9.6 ± 2.2
SVR (WU)			13.4 ± 6.4	12.7 ± 3.8
PVR (WU)	3.4 ± 1.1	4.6 ± 2.0**	4.0 ± 2.1	3.4 ± 1.8
LV stroke work (gm)	89 ± 28	128 ± 65*	74 ± 32	118 ± 48*
Carotid flow (mL/min)	272 ± 114	309 ± 132	202 ± 76	236 ± 64
Coronary flow velocity (cm/s)	19 ± 11	25 ± 14	22 ± 6	32 ± 17
NIRS head (%)	59 ± 5	55 ± 4	58 ± 4	58 ± 7
NIRS limb (%)	56 ± 4	55 ± 5	55 ± 6	55 ± 6

The table compares values before and after start of norepinephrine infusion in closed and opened fistula states.

Statistically significant results are displayed in bold. *p < 0.05, **p < 0.01, ***p < 0.001.

CO, cardiac output; Qa–arteriovenous fistula blood flow; COeff, effective cardiac output; HR, heart rate; SV, stroke volume; MAP–mean arterial pressure; PAMP, pulmonary artery mean pressure, SvO_2_—saturation of mixed venous blood with hemoglobin, TVR, total peripheral vascular resistance; SVR, systemic vascular resistance; PVR, pulmonary vascular resistance; LV, left ventricular; NIRS, near infrared spectroscopy.

Administration of norepinephrine expectably (Beurton et al., 2016) increased systemic vascular resistance during closed AVF. Surprisingly, there was no effect on TVR and SVR when the AVF was opened.

Norepinephrine significantly increased mean pulmonary artery pressure (from 26 ± 8 to 37 ± 14 mmHg, *p* < 0.01) and pulmonary vascular resistance (from 3.4 ± 1.1 to 4.6 ± 2.2 WU; *p* < 0.01) during closed AVF. The effect of norepinephrine on PAMP increase with opened AVF was less pronounced and lost statistical significance. However, the PVR insignificantly dropped after norepinephrine infusion.

Coronary artery flow increased by 31% after norepinephrine during with closed AVF and by 48% with opened AVF; the increase did not reach statistical significance in our small animal sample (*p* = 0.054 for closed and *p* = 0.1 for opened AVF). Carotid artery flow slightly rose after norepinephrine infusion in both fistula states, the difference was not statistically significant. Norepinephrine was not able to compensate the drop of carotid artery flow caused by opened arteriovenous fistula. NIRS values did not change after norepinephrine administration.

### Dobutamine

Dobutamine infusion led to a significant increase in cardiac output in both closed and open AVF (33% and 37%, respectively; *p* < 0.01 for both). The increase of cardiac output was driven by increase in heart rate and decrease in peripheral vascular resistance caused by systemic arterio-dilatation. Dobutamine did not affect mean systemic arterial pressure. Dobutamine decreased total vascular resistance, but its effect on systemic vascular resistance was blunted with AVF opened. Left ventricular stroke work was not significantly affected by dobutamine.

Mean pulmonary artery pressure rose with dobutamine, with unsignificant decrease in pulmonary vascular resistance.

Dobutamine increased the AVF flow (Qa) only by 4%. The Qa/CO ratio dropped significantly (from 24.4% ± 5.1% to 17.9% ± 3.8%; *p* < 0.01). The CO increase led to increase in effective cardiac output and thus in improvement of organ perfusion. COeff correlated well with MAP in opened AVF (R = 0.685, *p* = 0.061), but not during dobutamine administration (R = 0.416).

Coronary artery blood flow velocity increased in both closed and open AVF. Carotid artery blood flow increased significantly, improving the NIRS values measured on the head and on the front limb. However, the increase in head NIRS values was not significant with opened AVF.

Details are displayed in Table 2.

**TABLE 2:** Effect of continuous dobutamine infusion.

Parameter	Closed AVF		Opened AVF	
	Without dobutamine	With dobutamine	Without dobutamine	With dobutamine
CO (L/min)	7.8 ± 1.7	10.4 ± 2.7**	8.6 ± 3.6	11.8 ± 3.7**
Qa (L/min)			1.9 ± 0.3	2.0 ± 0.3
COeff (L/min)			6.6 ± 3.3	9.8 ± 3.4**
Qa/CO (%)			24.4 ± 5.1	17.9 ± 3.8**
HR (/min)	106 ± 30	159 ± 37**	126 ± 31	166 ± 45**
SV (mL)	78 ± 28	71 ± 31	71 ± 31	80 ± 47
MAP (mmHg)	83 ± 18	78 ± 23	74 ± 19	77 ± 21
PAMP (mmHg)	25 ± 7	30 ± 10*	25 ± 5	30 ± 8*
SvO_2_ (%)	59 ± 9	69 ± 11**	65 ± 9	70 ± 6*
TVR (WU)	10.9 ± 4.6	7.7 ± 3.3***	8.7 ± 4.0	6.5 ± 2.2*
SVR (WU)			12.2 ± 6.4	8.0 ± 2.8*
PVR (WU)	3.3 ± 0.9	2.9 ± 1.1	3.4 ± 1.4	2.7 ± 1.0
LV stroke work (gm)	90 ± 42	84 ± 52	75 ± 48	89 ± 66
Carotid flow (mL/min)	266 ± 104	383 ± 151**	199 ± 84	279 ± 122**
Coronary flow velocity (cm/s)	23 ± 15	36 ± 22*	25 ± 14	35 ± 20*
NIRS head (%)	59 ± 8	65 ± 8***	58 ± 10	60 ± 10
NIRS limb (%)	56 ± 5	59 ± 6*	54 ± 8	57 ± 7*

The table compares values before and after start of dobutamine infusion in closed and opened fistula states.

Statistically significant results are displayed in bold. *p < 0.05, **p < 0.01, ***p < 0.001.

CO, cardiac output; Qa–arteriovenous fistula blood flow; COeff, effective cardiac output; HR, heart rate; SV, stroke volume; MAP–mean arterial pressure; PAMP, pulmonary artery mean pressure, SvO_2_—saturation of mixed venous blood with hemoglobin, TVR, total peripheral vascular resistance; SVR, systemic vascular resistance; PVR, pulmonary vascular resistance; LV, left ventricular; NIRS, near infrared spectroscopy.

### Heart failure

The model of acute HF was defined as fast RV pacing leading to decrease of MAP under 60 mmHg. Necessary rate of pacing was insignificantly lower in open AVF than in closed AVF. Stroke volume substantially dropped during high heart rates. The open AVF had similar MAP decrease as the closed AVF. In both states, CO significantly dropped.

AVF blood flow dropped significantly with CO decrease, but Qa/CO ratio did not change. Therefore, the effective cardiac output decreased.

LV stroke work decreased during fast pacing in both opened and closed AVF

As a result of low systemic flow, carotid artery flow dropped. This was followed by a drop in NIRS both in limb and head, more pronounced during opened AVF.

Despite decreased CO, coronary artery flow velocity insignificantly increased after start of rapid pacing.

## Discussion

### Norepinephrine effect

In closed AVF condition, the effects of moderate dose norepinephrine (0.25 μg/kg/min) was largely in line with expectations: dominant increase in total vascular resistance (∼33%) and non-significant increase in cardiac output (∼10%) However, two distinct phenotypes of reaction to norepinephrine were identified: A) SVR increased by around 66% with a minor decrease in CO (8%) B) unchanged SVR but increase in cardiac output (20%).

We have no other explanation than the variable response of autonomic system to norepinephrine stimulation, since most other parameters such as pH, pCO_2_ and volemia were initially normal and neither were changing significantly throughout the observational period. This was an unexpected result. However, we occasionally see a similar pattern in other studies and these observations will deserve further attention.

In opened AVF condition, the response to norepinephrine was markedly different and surprising. Though the resulting effect is elevated arterial pressure (MAP +20%), the underlying mechanism here was a significant increase in cardiac output (∼36%), not in vascular resistance, which remained virtually unchanged. It was expected that norepinephrine would primarily enhance systemic vasoconstriction and thus divert blood flow from systemic circulation into the arteriovenous shunt, hence peripheral perfusion would be reduced. However, peripheral hemodynamic parameters changed insignificantly after norepinephrine infusion, specifically NIRS oximetries, carotid flow and coronary flow velocity—see Table 1.

Our results suggest a different scenario: Though, after norepinephrine, we did observe increased shunt flow (by ∼20%), disproportionally greater increase was seen in systemic cardiac output (∼36%). Since shunt resistance should essentially be constant (as AVF was modeled by relatively rigid cannulas), this implicates that systemic vascular resistance should decrease (which in fact happened in half of animals). On average we have seen no difference in SVR (relative to AVF without norepinephrine).

Increased cardiac output after norepinephrine in nearly all animals was achieved by increased stroke volume (by ∼40%) with one exception where the heart rate was the major driver. Increased stroke volume may again indicate considerable inotropic effect of norepinephrine. Relatively small increase in heart rate after norepinephrine administration may be explained by already elevated heart rate due to opened AVF. All of abovementioned changes implicate that myocardial stroke work and power were significantly increased.

At least two questions remain unanswered:

1. Reduced vascular resistance after noradrenaline. This was completely unexpected finding with possible, yet speculative explanations: One contributor can be a different sensitivity to alpha and beta norepinephrine stimulation among individuals, as discussed above, where some animals already in closed AVF phase of the protocol demonstrated no change in peripheral vascular resistance. This interindividual variability is not well documented, may be dose-dependent and further modified by propofol anesthesia (Biddle et al., 1995). Another mechanism can result from hyperkinetic circulation: norepinephrine combined with AVF represents high cardiac output state with increased flow velocities. Despite increased cardiac output neither pulmonary nor systemic resistances changed significantly. This is in contrast with norepinephrine alone where both systemic and pulmonary vascular resistance increased (see Table 1). Thus, in AVF + norepinephrine some global vasodilator activity must be present that may counteract vasoconstrictor effect of NE. Immediate drop in systemic vascular resistance after AVF opening has been described in past studies (Amerling et al., 2011).
2. Despite increased effective blood flow after norepinephrine administration local perfusion parameters, specifically NIRS and carotid flow remained generally unchanged. This seems contradictory, but in case of NIRS it may be explained by the adverse effect of norepinephrine on microcirculation, even in moderate doses and preserved cardiac output (Brassard et al., 2009). Regarding carotid flow, this is preferred circulation, so due to effective autoregulation the changes in flow may be blunted, specifically within hemodynamic conditions far from hemodynamic failure. Indeed, we have seen carotid flow increase in only three animals that reached lowest carotid flows after AVF opening. Others documented worsened splanchnic perfusion after norepinephrine (Priebe, Nöldge Gf Fau—Armbruster et al.).

### Dobutamine effect

From the systemic hemodynamics perspective, dobutamine seems valuable tool to increase systemic perfusion while not increasing systemic arterial pressure, nor shunt flow and not even myocardial work. Virtually all “additional” cardiac output recruited by dobutamine stimulation is available for peripheral perfusion as an effective CO. Indeed, carotid blood flow almost doubled.

There may be several clinical caveats regarding the effects of dobutamine:

### Myocardial perspective

In our experiment, dobutamine increased cardiac output almost exclusively by increasing the heart rate. Actually, stroke volume decreased in half of animals in closed AVF (n.s. for the whole group). This observation is in accordance with human study performed by Ahonen et al. (2008)—in infusion rate of 10 μg/kg/min dobutamine acts mostly by increasing heart rate without increasing (or even decreasing) stroke volume. Stroke work did not increase after dobutamine, but the myocardial power increased linearly with heart rate, i.e., by 30%. Increased heart rates over 150/min should not be limiting in young healthy pigs in terms of coronary flow (velocity increased by ∼50%) nor diastolic filling. However, we did see a strong negative correlation between heart rate and stroke volume (−0.840, *p* = 0.002) and this mechanism will certainly be a concern in polymorbid patients having an arteriovenous shunt.

Pulmonary artery pressure increased after dobutamine (both in closed and opened AVF), so unlike in systemic circulation, dobutamine had little if any effect on pulmonary vascular resistance.

From microcirculation perspective, relatively low improvements in peripheral oximetry (NIRS +3%) contrasts to massively increased perfusion (carotid flow doubled). This finding remains unclear. At least two alternative hypotheses exist: since peripheral perfusion was normal even before dobutamine, surplus of perfusion was diverted by peripheral autoregulation into tissue shunts to avoid excessive flow and shear in microvasculature; or dobutamine itself can interfere with peripheral autoregulation again resulting in opening local shunts. Interestingly, in the study by Priebe et al. (1995) dobutamine had neutral effect on splanchnic perfusion in pigs.

Lastly, we did not observe hypotension after dobutamine. However, in case it develops, vasopressors administration could increase shunt flow since shunt fraction is linearly proportional to systemic vascular resistance (R = 0.85). Thus, the desire to increase peripheral flow and reduce shunt fraction could be negated, at least in part.

### Comparing the effects of norepinephrine and dobutamine

Dobutamine increased CO without affecting mean arterial pressure and thus acted primarily via systemic arterio-dilatation and by heart rate increase. It did not affect LV work. Dobutamine also did not increase AVF flow; rise of CO was therefore primarily by opened peripheral resistant arterioles lead to rise in organ perfusion (carotid artery, coronary artery).

Norepinephrine increased mean arterial pressure and CO; increase in CO was lower than in dobutamine (+2.2 L/min for norepinephrine, +3.6 L/min in dobutamine, *p* = 0.08). Norepinephrine also increased both AVF flow and effective CO, but at the cost of increased LV work. The increase in AVF flow was significantly related to increased MAP. Increase in organ perfusion (carotid artery, coronary artery) was not statistically significant.

### Heart failure and AVF

In opened AVF condition cardiac output of at least 6 L/min was necessary to keep MAP within 40–60 mmHg. Since 22%–33% (∼1.6 L/min) were shunted by AVF, the effective cardiac output was ∼4.5 L/min, similar to cardiac output in our closed AVF condition. Even under these circumstances (CO 40% of baseline, MAP 40–50 mmHg) we have seen normal coronary flow velocities and acceptable microcirculation (NIRS over 55%). Due to the shunt flow, pulmonary perfusion, saturation of mixed venous blood and pulmonary artery pressure were nearly normal probably not giving the reason for increased intrapulmonary shunting, hypoxia and further deterioration.

Flow through the AVF is predictable even in low CO state, since Qa also correlated with mean arterial pressure in all animals (R = 0.781). Additionally, unlike in most other situations presented in this report, at very low pressures (MAP <55 mmHg) also effective cardiac output correlated very well with the systemic pressure (R = 0.920). This indicates that the systemic resistance is constant, possibly approaching its limits. Indeed, in these conditions we have observed lowest resistances (∼8.2 WU). Administering vasopressors in such situation could again be quite counterproductive since increased systemic vascular resistance would divert already poor systemic perfusion into the AV shunt.

The comparison of tachycardic heart failure with and without shunt conditions (Table 3) brings further interesting observations:

1. Though the macrohemodynamic parameters were nearly identical in opened AVF vs. closed AVF, (effective CO ∼4.8 L/min, i.e., 40 % of baseline; MAP ∼50 mmHg; HR∼ 200/min; coronary flow velocity ∼24 cm/s), peripheral oximetry (NIRS_head_) was significantly lower in the opened AVF condition (56% vs. 61%, *p* = 0.01). Whether this reflects compromised autoregulation as suggested above, remains to be elucidated.
2. During rapid pacing, total cardiac output decreased substantially less in AVF condition (by∼1.8 L/min, i.e., 20 %) than in closed AVF (by 2.8 L/min, i.e., 40 %). This occurred despite the induction was nearly identical, in most cases with the heart rate same for both conditions. Thus, mainly stroke volume decrease was responsible for the difference in cardiac output. Hypothetically, AVF circulation provides better preload and lower afterload, that might be one explanation of the stroke volume difference. This observation also requires further exploration.

**TABLE 3:** Effect of low cardiac output.

Parameter	Closed AVF		Opened AVF	
	Without heart failure	With heart failure	Without heart failure	With heart failure
CO (L/min)	8.0 ± 1.3	4.8 ± 1.2***	8.1 ± 0.9	6.3 ± 0.9*
Qa (L/min)			2.1 ± 0.3	1.6 ± 0.2***
COeff (L/min)			6.1 ± 1.0	4.7 ± 0.8*
Qa/CO (%)			25.2 ± 4.7	25.2 ± 2.4
HR (/min)	136 ± 32	216 ± 57**	127 ± 27	194 ± 58*
SV (mL)	61 ± 15	24 ± 8***	67 ± 14	37 ± 11**
MAP (mmHg)	89 ± 14	51 ± 6***	67 ± 11	48 ± 9**
PAMP (mmHg)	24 ± 10	24 ± 6	26 ± 7	24 ± 7
SvO_2_ (%)	59 ± 13	44 ± 15***	59 ± 11	53 ± 13*
TVR (WU)	10.6 ± 1.9	9.4 ± 2.5*	7.7 ± 2.1	6.1 ± 0.7
SVR (WU)			10.5 ± 3.6	8.2 ± 0.9
PVR (WU)	3.2 ± 1.7	5.3 ± 1.7**	3.3 ± 1.0	4.0 ± 1.4
LV stroke work (gm)	76 ± 27	16 ± 6***	60 ± 11	23 ± 10**
Carotid flow (mL/min)	280 ± 110	189 ± 110***	217 ± 78	140 ± 67**
Coronary flow velocity (cm/s)	25 ± 12	28 ± 14	24 ± 9	24 ± 9
NIRS head (%)	64 ± 9	61 ± 8	61 ± 10	56 ± 10*
NIRS limb (%)	59 ± 5	57 ± 3	56 ± 5	53 ± 6*

The table compares values before and after start of dobutamine infusion in closed and opened fistula states.

Statistically significant results are displayed in bold. *p < 0.05, **p < 0.01, ***p < 0.001.

CO, cardiac output; Qa–arteriovenous fistula blood flow; COeff, effective cardiac output; HR, heart rate; SV, stroke volume; MAP–mean arterial pressure; PAMP, pulmonary artery mean pressure, SvO_2_—saturation of mixed venous blood with hemoglobin, TVR, total peripheral vascular resistance; SVR, systemic vascular resistance; PVR, pulmonary vascular resistance; LV, left ventricular; NIRS, near infrared spectroscopy.

## Limitations

The chosen AVF model is robust, safe and provides consistently high shunt flow. However, it includes rigid cannulas, and thus has constant resistance and lacks ability to collapse at very low flows. However, it is known that AVF resistance is regulated mainly by the arterial anastomosis (entry site) (Sullivan et al., 1993). Cannulas were inserted directly into central vessels while typical hemodialysis AVF is more peripherally located (Fila, 2021). Most importantly, it lacks maturation over weeks/months, that would typically take fistula to evolve into high flow shunt, and subsequential long-term hemodynamic changes. Our HF model may well represent acute tachycardic event, but care should be taken when translating the results into other forms of heart failure, specifically chronic forms. Usually, few weeks of rapid pacing is required for full development of hemodynamic and neurohumoral changes typical for heart failure (Cops et al., 2019). In this study we only aimed at redistribution of acutely decreased cardiac output.

From translational medicine perspective this model has inherited limitations of most animal models, such as young animals free of comorbidities with potent autoregulatory mechanism. Typical metabolic rate of adolescent swine is 150%–200% relative to adult human of the same weight. Thus, also most hemodynamic parameters in our subjects are 1.5-2x higher than in adult humans making direct comparisons less intuitive. Known hemodynamic difference in swine to humans is highly sensitive pulmonary vasoconstriction resulting in frequent pulmonary hypertension. Frequently we observe significant cardiotonic effect of norepinephrine that may be uncommon in adult humans. We planned to study the effects of norepinephrine and dobutamine also in the heart failure model. However, administration of these drugs caused fast development of ventricular fibrillation.

## Conclusion

We have extensively explored challenging, clinically highly relevant hemodynamic conditions of AVF. Under all circumstances, AVF flow is proportional to systemic perfusion pressure and thus well predictable. However, the ratio of shunt flow to systemic flow depends on systemic vascular resistance which is less easily available. Significantly increased resistance might contribute to stealing effect of AVF. Norepinephrine in our model and given dose elevated blood pressure primarily by increasing effective cardiac output, at the cost of increased myocardial work and power. Surprisingly, vascular resistance was not increased significantly, so the steal effect of the AV shunt was not aggravated.

Dobutamine increased effective cardiac output but not systemic pressure, by combined effect of elevated cardiac output and vasodilatation. The shunt fraction was thus reduced. Stroke work did not change, but myocardial power increased significantly, proportionally to the rise in heart rate. Heart rate (and probably also the systemic vasodilatation) were the sole drivers of increased CO.

In low cardiac output, AVF contributed to dramatic decrease in arterial pressure. However, attempts to increase peripheral vascular resistance might increase shunt flow without improving organ perfusion.

The results of our porcine study showed that the impact of a high-flow AVF on hemodynamics in these two extreme situations is significant. We assume that this situation may be similar in patients with high-flow fistulas.

## Data Availability

The raw data supporting the conclusion of this article will be made available by the authors, without undue reservation.

## Abbreviations

AVF, arteriovenous fistula; CVP, central venous pressure; CO, cardiac output; Coeff, effective cardiac output; ECG, electrocardiogram; EtCO_2_, end-tidal carbon dioxide; HR, heart rate; i.m., intramuscular; i.v., intravenous; MAP, mean arterial pressure; PAMP, pulmonary artery mean pressure; pCO_2_, partial pressure of carbon dioxide; PCWP, pulmonary capillary wedge pressure; pO_2_, partial pressure of oxygen; PVR, pulmonary vascular resistance; rSO_2_, regional oxygen saturation; SvO_2_, venous oxygen saturation; SVR, systemic vascular resistance; TVR, total vascular resistance; WU, Wood units.
